# The Insula Is a Hub for Functional Brain Network in Patients With Anti-*N*-Methyl-D-Aspartate Receptor Encephalitis

**DOI:** 10.3389/fnins.2021.642390

**Published:** 2021-03-15

**Authors:** Chunyan Li, Xiaomin Pang, Ke Shi, Qijia Long, Jinping Liu, Jinou Zheng

**Affiliations:** ^1^Department of Neurology, The First Affiliated Hospital of Guangxi Medical University, Nanning, China; ^2^Department of Radiology, The First Affiliated Hospital of Guangxi Medical University, Nanning, China

**Keywords:** anti-*N*-methyl-D-aspartate receptor encephalitis, resting-state functional MRI, graph theory, Granger causality analysis, cognitive impairment

## Abstract

**Background:**

In recent years, imaging technologies have been rapidly evolving, with an emphasis on the characterization of brain structure changes and functional imaging in patients with autoimmune encephalitis. However, the neural basis of anti-*N*-methyl-D-aspartate receptor (NMDAR) encephalitis and its linked cognitive decline is unclear. Our research aimed to assess changes in the functional brain network in patients with anti-NMDAR encephalitis and whether these changes lead to cognitive impairment.

**Methods:**

Twenty-one anti-NMDAR encephalitis patients and 22 age-, gender-, and education status-matched healthy controls were assessed using resting functional magnetic resonance imaging (fMRI) scanning and neuropsychological tests, including the Hamilton Depression Scale (HAMD_24_), the Montreal Cognitive Assessment (MoCA), and the Hamilton Anxiety Scale (HAMA). A functional brain network was constructed using fMRI, and the topology of the network parameters was analyzed using graph theory. Next, we extracted the aberrant topological parameters of the functional network as seeds and compared causal connectivity with the whole brain. Lastly, we explored the correlation of aberrant topological structures with deficits in cognitive performance.

**Results:**

Relative to healthy controls, anti-NMDAR encephalitis patients exhibited decreased MoCA scores and increased HAMA and HAMD_24_ scores (*p* < 0.05). The nodal clustering coefficient and nodal local efficiency of the left insula (Insula_L) were significantly decreased in anti-NMDAR encephalitis patients (*p* < 0.05 following Bonferroni correction). Moreover, anti-NMDAR encephalitis patients showed a weakened causal connectivity from the left insula to the left inferior parietal lobe (Parietal_Inf_L) compared to healthy controls. Conversely, the left superior parietal lobe (Parietal_sup_L) exhibited an enhanced causal connectivity to the left insula in anti-NMDAR encephalitis patients compared to controls. Unexpectedly, these alterations were not correlated with any neuropsychological test scores.

**Conclusion:**

This research describes topological abnormalities in the functional brain network in anti-NMDAR encephalitis. These results will be conducive to understand the structure and function of the brain network of patients with anti-NMDAR encephalitis and further explore the neuropathophysiological mechanisms.

## Introduction

Anti-*N*-methyl-D-aspartate receptor encephalitis (NMDAR) is an autoimmune disease mediated by IgG antibodies to the NR1 subunits of NMDA receptors. The disease, which is regarded as the most common type of autoimmune encephalitis, was first discovered in 2007 by Dalmau and colleagues (Dalmau et al., 2007). Patients usually present with mental and behavioral alterations, cognitive disorders, near memory loss, seizures, speech disorder, dyskinesia, involuntary movement, consciousness disorders, and autonomic nervous dysfunction (Dalmau et al., 2008). Accurate diagnosis of the disease remains difficult due to its atypical symptoms, and there is a tendency for it to be confused with other neurological disorders. The majority of patients with anti-NMDAR encephalitis have normal brain magnetic resonance imaging (MRI) imaging findings. A recent study suggests that approximately 50% of patients require intensive care unit (ICU) admission, while 5.6% of patients die (Deng et al., 2019). Moreover, patients are regularly left with permanent neurologic disability even after recovery, especially in cognitive domains such as memory, attention, and executive control (McKeon et al., 2018). Such cognitive problems may result in a significant impact on the lives and social interactions of (recovered) anti-NMDAR encephalitis patients. However, little is known about the pathophysiological mechanisms of persistent anti-NMDAR encephalitis in patients with cognitive impairment. Following the discovery of anti-NMDAR receptor encephalitis, many neuroimaging techniques have been used for researching underlying pathological factors, such as positron emission tomography (PET) (Leypoldt et al., 2012), functional magnetic resonance imaging (fMRI), and electroencephalography (EEG) (Schmitt et al., 2012). In particular, functional magnetic resonance has been used to study the neuropathological and physiological mechanisms implicated in anti-NMDAR encephalitis patients (Finke et al., 2013; Peer et al., 2017; Cai et al., 2020). However, few studies have so far explored anti-NMDAR encephalitis-elicited alterations of functional brain networks base on graph theory. In recent years, graph theory has been widely used in neuropsychological imaging (Achard et al., 2006; delEtoile and Adeli, 2017; Zhou et al., 2019). In graph theory, a network constructed by nodes and edges is measured by numerous properties to evaluate network separation and integration effects (Stam and Reijneveld, 2007; Rubinov and Sporns, 2010). In addition, to investigate directed junction networks involved in patients with anti-NMDAR encephalitis, Granger causality analysis (GCA) can be used to evaluate the causal connectivity between time series recorded at the same time (Zang et al., 2012). GCA is a reliable method for assessing causal association which can study directional connections between different brain regions and dynamically observe changes in brain networks. It has been used in several studies examining the pathogenesis of neurodegenerative diseases (Li et al., 2019; Pang et al., 2019; Hao et al., 2020). In our present research, we used graph theory to quantify the topological properties of the brain network in anti-NMDAR encephalitis patients to elucidate the network nodes and causal connectivity of brain function. Based on the aberrant topological organization, we used GCA to analyze the causal relationship between these changes and the whole brain. Furthermore, we aimed to determine whether aberrant topological organization and causal connection abnormalities are related to cognitive dysfunction performance in anti-NMDAR encephalitis patients.

## Materials and Methods

### Participants

Twenty-one anti-NMDAR encephalitis patients who met the diagnostic criteria of anti-NMDAR encephalitis by Graus et al. (2016) were recruited from inpatients or outpatients at the Department of Neurology of the First Affiliated Hospital of Guangxi Medical University between March and December 2017 after the acute stage of the disease, and 21 gender-, age-, and education status-matched healthy controls (HC) were recruited from the community, excluding subjects with neurological or psychiatric disease. The acute, subacute, or recovery phases of anti-NMDAR encephalitis patients were defined as 3, 3–12, and > 12 months after initial immunotherapy. Inclusion criteria for anti-NMDAR encephalitis patients were as follows: (Dalmau et al., 2007) presentation with one or more of the following six major symptoms: behavioral disorders or cognitive impairments, speech disorders, seizures, motor disorders or involuntary movements, decreased consciousness levels, and autonomic dysfunction or central hypoventilation; (Dalmau et al., 2008) anti-NMDAR antibodies in cerebrospinal fluid or serum (Deng et al., 2019). All patients were right-handed and between 18 and 60 years old. Exclusion criteria were as follows: (Dalmau et al., 2007) patients exhibited concurrent severe physical illness, substance abuse, or non-cooperation in examination; (Dalmau et al., 2008) abnormalities in structural MRI images. All patients and control subjects provided written informed consent before participation in the study. The Hamilton Anxiety Rating Scale (HAMA) and Hamilton Depression Rating Scale (HAMD_24_) scoring were carried out to evaluate anxiety and depression in all participants, while the Montreal Cognitive Assessment (MoCA) was used to measure the cognition. All subjects underwent an MRI scan. The study protocol was approved by the First Affiliated Hospital of Guangxi Medical University Ethics Committee.

### MRI Data Acquisition

Magnetic resonance imaging data were acquired using an Achieva 3.0-T MRI scanner with a 12-channel head coil (Philips, Amsterdam, Netherlands). Prior to scanning, each subject was asked to rest for 20 min. During MRI scanning, subjects were instructed to close their eyes, remain conscious, and avoid thinking about anything. Foam padding was utilized for noise mitigation and limitation of head movements. For each subject, resting-state functional imaging was obtained using the echo-planar image (EPI) technique with the following parameters: repetition time (TR) = 2,000 ms, echo time (TE) = 30 ms, 31 slices and 180 volumes, slice thickness = 5 mm, slice gap = 1 mm, voxel size = 3.44 mm × 3.44 mm × 6.00 mm, field of view = 220 mm × 220 mm, flip angle = 90°, and scanning time = 360 s.

### Resting-State fMRI Data Preprocessing

Pre-processing of functional images was carried out using GRETNA^1^ based on statistical parametric mapping (SPM12)^2^ in MATLAB 2013b (Wang et al., 2015). All DICOM files were converted into NifTI files. The first 10 volumes were discarded for promotion in magnetic field stability and reduction of artifacts. Slice-timing was conducted for correction of time differences between slices, and realignment was conducted for correction of head motion. Patients with a head motion of > 2 mm or head rotations < 2° were excluded from further analyses. Volumes were normalized to the Montreal Neurological Institute (MNI) (Tzourio-Mazoyer et al., 2002) space with a resampling resolution of 3 mm × 3 mm × 3 mm, and the data were smoothed with a Gaussian kernel of 6 mm × 6 mm × 6 mm full width at half maximum (FWHM) Gaussian kernel. The data were temporally detrended, and nuisance covariates including signal from white matter, cerebrospinal fluid, global signal, and Friston 24 parameters were regressed out. Finally, all images were bandpass-filtered (0.01–0.08 Hz).

### Network Construction and Analysis

The GRETNA toolbox was used to construct a functional network composed of nodes and edges with sparsity ranging from 10 to 40% and connection density interval of 0.01, and automatic anatomical landmark (AAL) template was used to construct a 90 × 90 temporal correlation matrix of the whole brain (Gong et al., 2009; Lei et al., 2015). To describe the topology of patients with anti-NMDAR encephalitis, we calculated the characteristics of the topological network including small-world, global/local efficiency, degree centrality, intermediate centrality, and node efficiency based on the constructed brain network (Rubinov and Sporns, 2010). To determine if there were significant group differences in the topographic parameters (including C_*p*_, C_*l*_, E_*glob*_, E_*nod*_, N_*Deg*_, and N_*Bet*_), we used the two-sample *t* test analysis on the area under curve (AUC) of all metrics with age, gender, and education as covariates. To address the problem of multiple comparisons of nodal centralities, we conducted Bonferroni correction (*p* < 0.05 after Bonferroni correction was deemed statistically significant).

### Granger Causal Connectivity Analysis

The basic approach of GCA was to explore two discrete time series, such as X and Y. For time series X and Y, if adding the past information of Y is better than simply using the past information of X to predict X, then it is said that Y contributes to X, and there is a Granger causality from Y to X. The GCA coefficient was used to evaluate the Granger causality. An increase in GCA may imply excitatory effects or a positive feedback, while a decrease in GCA value may imply inhibitory effects or a negative feedback (Blinowska et al., 2004; Chen et al., 2009). Brain regions with significant differences between the two groups were then identified as regions of interest (ROIs). In this study, we performed GCA using the RESTPLUS^3^ software package with the left insula (Insula_L) as the seed ROI (Jia et al., 2019). The time sequence of Insula_L was defined as the seed time sequence X, and time sequence Y represented the time sequence of all voxels in the brain. Then we calculated the linear direct effects from X to Y and Y to X using voxels from the entire brain. Thus, for each participant, a Granger causality diagram from the Insula_L to the whole brain and the whole brain to the Insula_L based on the impact metric for each subject was obtained. These Granger causality maps were subsequently analyzed using two-sample *t* test with SPM12. Statistical significance was determined by AlphaSim correction, with a voxel-level significance threshold of *p* < 0.001 and cluster-level *p* < 0.05 (determined by a Monte Carlo simulation). Age, gender, and education level were applied as covariates to minimize their potential effects on the analysis.

### Statistical Analysis

Clinical data, head motion, and neuropsychological test scores were analyzed using SPSS 23.0 (SPSS Inc., Chicago, IL, United States). First, the Kolmogorov–Smirnov test was conducted to determine whether the quantitative data conforms to a normal distribution. Second, inter-group comparisons were performed according to the normality of data: data with normal distribution were statistically analyzed by independent *t* test, while data with non-normal distribution were examined by Mann–Whitney *U* test. Gender, a binary variable, was assessed by the chi-square test. *p* < 0.05 indicated statistically significant difference.

The mean nodal clustering coefficient values, nodal local efficiency values of the left insula, and GCA values of brain regions with statistical discrepancy were extracted for correlation analysis with neuropsychological scores by Pearson’s (normal distribution data) or Spearman’s (non-normally distributed data) method. *p* < 0.05 indicated statistically significant difference.

### Network Visualization

The results of functional networks were displayed using the Brainnet Viewer (Xia et al., 2013).

## Results

### Demographic, Head Motion, and Clinical Characteristics

No significant differences was found for age, gender, years of education, and head motion between anti-NMDAR encephalitis patients and HCs (*p* > 0.05). Compared with HCs, anti-NMDAR encephalitis patients showed a significantly poorer performance in neuropsychological test scores including the MoCA, HAMA, and HAMD_24_ (*p* < 0.05). The clinical and epidemiological details are summarized in Table 1.

**TABLE 1:** Comparison of clinical data and neuropsychological scores between the two groups.

Characteristics	Patients	Controls	*p* value
Gender (M/F)	11/10	10/11	0.758^*a*^
Age (years)	28.14 ± 10.20	29.38 ± 6.078	0.635
Education (years)	13.10 ± 3.477	14.10 ± 3.192	0.337
HAMD_24_ scores	5.76 ± 6.449	0.52 ± 0.981	0.003
HAMA scores	4.67 ± 3.706	0.86 ± 1.014	<0.001
Executive function	0.62 ± 0.498	1.00 ± 0.000	0.002
Fluency	0.95 ± 0.669	1.81 ± 0.512	<0.001
Orientation	5.57 ± 1.165	6.00 ± 0.000	0.107
Calculation	2.67 ± 0.658	3.00 ± 0.000	0.031
Abstraction	2.05 ± 0.973	2.95 ± 0.218	<0.001
Delayed recall	2.81 ± 1.662	4.33 ± 0.913	0.001
Visual perception	2.43 ± 0.746	3.00 ± 0.000	0.002
Naming	3.90 ± 0.301	4.00 ± 0.000	0.162
Attention	2.86 ± 0.478	2.81 ± 0.402	0.729
MoCA total score	23.86 ± 3.966	28.90 ± 1.221	<0.001
Head motion	0.12 ± 0.05	0.09 ± 0.03	0.032

**p* < 0.05 indicates statistical significance. ^*a*^χ^2^ test for sex (*n*). Independent *t*-test for the other data (means ± SD).*

### Altered Nodal Strength in the Functional Network

Compared to matched random networks, both anti-NMDAR encephalitis patients and HCs exhibited characteristics of a small-world network. No significant differences in global properties were found between the patients and HCs. To study alterations in nodal strength in the anti-NMDAR encephalitis patients, we analyzed the characteristics of several nodal characteristics. The nodal clustering coefficient and the nodal local efficiency of the left insula in anti-NMDAR encephalitis patients were significantly lower compared to controls (*p* < 0.05 following Bonferroni correction; Table 2).

**TABLE 2:** Nodes of significant abnormal topological alterations in functional networks between the two groups.

Nodal metric	Node	Patients	HCs	*p* value
Nodal clustering coefficient	INS.L	0.179 ± 0.050	0.238 ± 0.032	<0.001
Nodal local efficiency	INS.L	0.223 ± 0.060	0.282 ± 0.026	<0.001

*INS, insula; L, left; R, right. Bonferroni correction (*p* < 0.05).*

### GCA Results

**FIGURE 1:**
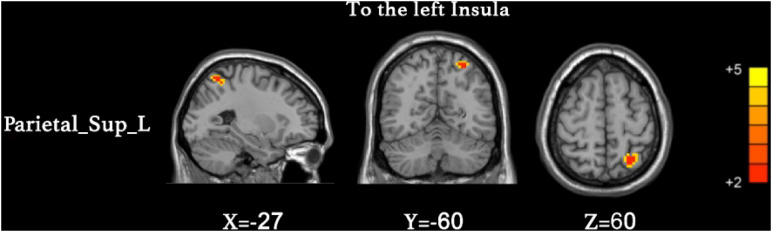
Increased causal connectivity from the superior parietal lobe (Parietal_sup_L) to the left insula in anti-NMDAR encephalitis patients. The red color represents an increased causal connectivity. AlphaSim correction with a voxel-level significance threshold of *p* < 0.001 and cluster-level *p* < 0.05.

**FIGURE 2:**
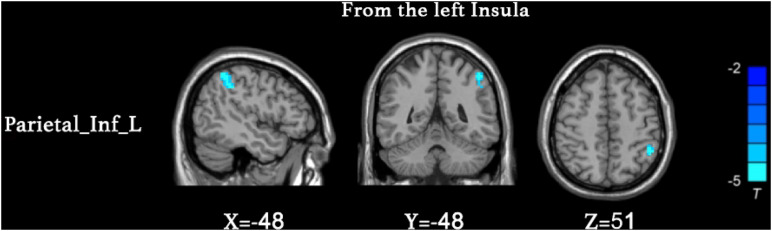
Decreased causal connectivity from the left insula to the inferior parietal lobe (Parietal_Inf_L) in anti-NMDAR encephalitis patients. The blue color represents a decreased causal connectivity. AlphaSim correction with a voxel-level significance threshold of *p* < 0.001 and cluster-level *p* < 0.05.

We found that the superior parietal lobe (Parietal_sup_L) exhibited enhanced causal connectivity to the left insula compared to controls (Table 3 and Figure 1). The estimated smoothness of the GCA map was FWHMx = 10.092 mm, FWHMy = 10.554 mm, FWHMz = 10.844 mm. Compared to the HCs, the anti-NMDAR encephalitis patients manifested a weakened causal connectivity from the left insula to the Parietal_Inf_L (Table 4 and Figure 2). The estimated smoothness of the GCA map was FWHMx = 10.026 mm, FWHMy = 10.841 mm, FWHMz = 10.831 mm.

**TABLE 3:** The causal connectivity from the Parietal_sup_L to the Insula_L between the two groups.

Seed region	Comparison	Brain region	BA	Peak MNI			*t* value	Voxels
	
				X	Y	Z		
INS_L	HC < PA	Parietal_sup_L	7	−27	−60	60	3.3256	53

*INS, insula; Parietal_sup, Parietal_superior; BA, Brodmann’s area; MNI, Montreal Neurological Institute; L, left; R, right. AlphaSim correction with a voxel-level significance threshold of *p* < 0.001 and cluster-level *p* < 0.05.*

**TABLE 4:** The causal connectivity from the Insula_L to the Parietal_Inf_L between the two groups.

Seed region	Comparison	Brain region	BA	Peak MNI			*t* value	Voxels
	
				X	Y	Z		
INS_L	HC > PA	Parietal_Inf_L	40	−48	−48	51	3.3256	48

*INS, insula; Parietal_Inf, Parietal_inferior; BA, Brodmann’s area; MNI, Montreal Neurological Institute; L, left; R, right. AlphaSim correction with a voxel-level significance threshold of *p* < 0.001 and cluster-level *p* < 0.05.*

### Correlation Analysis

There were no significant correlations between aberrant topological properties values or GCA values in brain regions and MoCA total scores, HAMA, and HAMD_24_ scores (*p* > 0.05).

## Discussion

**FIGURE 3:**
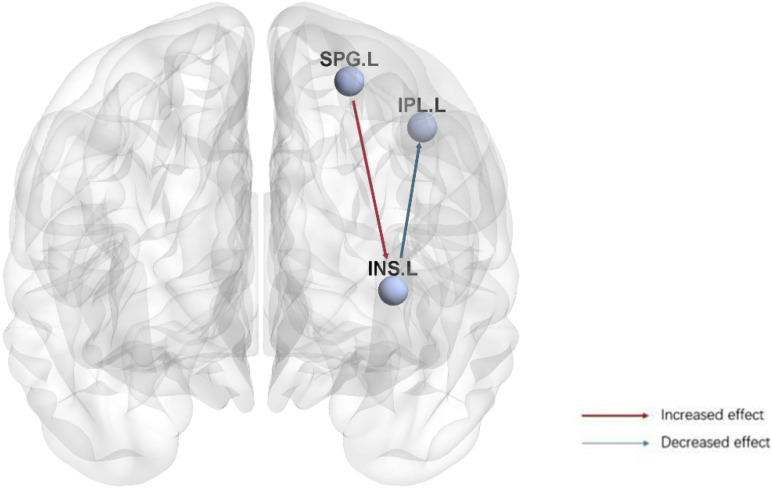
Visualization of the causal connectivity from the left insula to the inferior parietal lobe (Parietal_Inf_L) and from the superior parietal lobe (Parietal_sup_L) to the left lnsula in anti-NMDAR encephalitis patients. The red arrow indicates an increased causal relationship, and the blue arrow indicates a decreased causal relationship. Bonferroni correction (*p* < 0.05). (SPG.L, Parietal_sup_L; IPL.L, Parietal_Inf_L; INS.L, Insula_L).

This study utilized both graph theory and GCA to investigate the functional brain network and causal connectivity in anti-NMDAR encephalitis patients compared to controls. Using graph theory analysis, we found the nodal clustering coefficient and nodal local efficiency of the left insula in anti-NMDAR encephalitis patients were significantly decreased compared to controls. In GCA analysis, we selected the left insula to explore its causal effects both from and to the whole brain (Figure 3). Specifically, compared to healthy controls, anti-NMDAR encephalitis patients showed weakened causal connectivity of the left insula with the inferior parietal lobe (Parietal_Inf_L). Conversely, the superior parietal lobe (Parietal_sup_L) exhibited an enhanced causal connectivity with the left insula in anti-NMDAR encephalitis patients. Unexpectedly, our results showed that these alterations were not associated with any neuropsychological test scores. The current study may provide deeper insights into the aberrant brain network of anti-NMDAR encephalitis patients and further explore potential underlying neuroimaging mechanisms.

Graph theory studies the organization of the entire brain network (Bullmore and Sporns, 2009; He and Evans, 2010; Bullmore and Bassett, 2011). In a normal brain network, a high clustering coefficient and local efficiency are parameters reflecting high local specialization of information processing. Conversely, low path lengths and high global efficiency represent a powerful ability to integrate information from the entire brain (He et al., 2009; Vlooswijk et al., 2011; Reijmer et al., 2013). The optimal brain network combines the functions of balanced separation and information processing integration. Results from patients with anti-NMDAR encephalitis suggest that a reduced clustering coefficient and reduced local efficiency are characteristics of more random networks, which can be interpreted as the loss of network organization.

The insula is the only lobe hidden in the deep regions of the cerebral hemisphere, and there has so far been only little research into its function. Although the function of the insula is therefore still not entirely clear, it has been suggested to be related to memory, visceral sensations, affection, addiction, pain regulation, language, and cardiac activity (Chang et al., 2013). More recently, the insula has been suggested to represent a hub (e.g., for the central executive and default mode networks) for dynamic switching between different networks. Serving as an essential intermediate hub for many different brain regions, it may help to access attention, working memory, and other higher-order cognitive processes (Menon and Uddin, 2010; Chang et al., 2013). It has been reported that the cognitive decline of many diseases may be related to the faulty connectivity of insular networks (Xie et al., 2012; Christopher et al., 2014). Therefore, we speculated that the impairment of cognitive function in patients with anti-NMDAR encephalitis may be related to an abnormal topology of the insula. Recent evidence suggests that insular activity may be a harbinger of cognitive processes, with insular activity being particularly associated with the central executive network [the dorsolateral prefrontal cortex (DLPFC), the posterior parietal cortex (PPC), and the default mode network (DMN)] (Seeley et al., 2007; Sridharan et al., 2008). In particular, the central executive network helps to maintain and manipulate information in the working memory as well as make judgements and decisions about various types of information. The insula selectively initiates the recruitment of brain regions relevant to the task at hand to govern cognitive performance. The nodal clustering coefficient and nodal local efficiency of the left insula represent a loss in network organization and renders the above-mentioned network switching tasks difficult, which in turn may lead to cognitive dysfunction in anti-NMDAR encephalitis patients.

Granger causal connectivity is a method that, from region A to another region B, proves how neuronal activity in A predicts activity in B. Causal effective functional connectivity has been observed between the left insula and the parietal lobe. As one of the primary structures of the human brain, the parietal lobe exerts important physiological functions closely associated to perception, calculation, attention, episodic recall, and spatial cognition (Corbetta and Shulman, 2002; Sack et al., 2002; Dehaene et al., 2004; Cabeza et al., 2008). At the same time, the neural network theory suggests that cognitive functions are not limited to a certain lobe or functional area of the brain—instead, there are extensive and close connections between areas of the brain. The left insula to whole brain GCA showed that the left insula activity predicted a decrease in follow-up activity in the inferior parietal lobe. From our research results, we speculate that an abnormal insular function leads to a subsequent reduction in parietal function. Severe cognitive dysfunction (such as apraxia, agnosia, miscalculation, and situational memory impairment) may occur after parietal lobe damage, with or without varying degrees of aphasia. It is worth noting that research on functional brain imaging has shown that human hippocampal information has a strong functional connection with the ventral parietal lobe (Vincent et al., 2006). The ventral parietal lobe may use the same method to process sensory information and memory information. It has been reported that hippocampal NMDAR function is impaired (Peer et al., 2017) and may be related to the memory deficit of patients. We speculated that changes in the connectivity between the parietal lobe and the insula were related to memory impairment. Our findings emphasized that impaired parietal NMDAR function is related to the pathophysiology and neural mechanisms of memory deficits in these patients. At the same time, the whole brain to the left insula GCA also revealed that anti-NMDAR encephalitis patients had remarkably enhanced causal connectivity from the superior parietal lobe to the left insula, suggesting that activity in the left superior parietal lobe may predict increases in activity in the left insula. Patients with anti-NMDAR encephalitis show a cognitive decline after the onset of the disease. Based on previous reports, various cognitive functions of patients with encephalitis improve to varying degrees in the late recovery period, with obvious cognitive functional recovery in the first year after the illness (Chen et al., 2018; McKeon et al., 2018). However, in some patients, cognitive symptoms do not fully subside. Therefore, we speculated that prolonged symptoms may be related to compensation mechanisms for the enhanced dorsal parietal lobe activity or may be caused by damaged glial cells during the recovery period, although further pathological studies are needed. Our results suggest that insular functional connectivity is impaired, which in turn affects the function of the superior parietal lobe, thus interfering with the maintenance, transmission, and feedback of information, leading to a decline in the patients’ cognitive ability. In addition, neuroanatomic lesions of cognitive control processes include the insula and DLPFC as well as the lateral and medial parietal frontal cortex (Walter et al., 2016). Previous studies described above indicated that the structural and functional alterations in the insula and parietal lobes may be one of the potential mechanisms underlying the impaired cognitive function in patients with anti-NMDAR encephalitis.

In this study, we found that in fact, these alterations were not related to either MoCA, HAMA, or HAMD_24_ scores. The following are several reasons we consider possible for this: first, the sample size (only 21 cases) of our study was limited. Second, all anti-NMDAR encephalitis patients in our study were in the convalescence phase; therefore, patients’ cognitive functions may have recovered to varying degrees (McKeon et al., 2018). More longitudinal studies involving patients at different stages of the recovery process may therefore be required in the future. Third, the MoCA, HAMA, and HAMD_24_ scales are subjective and may not be sensitive enough to fully identify differences in patient recovery. Thus, it is necessary to use more sensitive neuropsychological tools to evaluate cognitive performance indicators and conduct multi-faceted research in the future.

Our study also had some limitations. First, our experimental design was cross-sectional, and as discussed, the sample size was limited. Additional longitudinal studies with a larger sample size and involving patients at different stages of recovery are therefore required in the future. Second, as patients with acute anti-NMDAR encephalitis were largely non-cooperative in examination we recruited patients in the convalescence phase with different courses, who also exhibited differences in brain function. Therefore, the interpretations of our findings do not fully represent changes in patients at a certain stage. Third, the optimal analysis strategy for graph theory remains controversial. Therefore, in future studies, multimodal MRI should be used to reveal abnormalities of brain structure and function, so as to deepen our understanding of the neuropathological and physiological mechanisms of cognitive impairment of anti-NMDAR encephalitis.

## Conclusion

To sum up, previous studies indicated abnormal functional network centers in several regions of the brain in anti-NMDAR encephalitis patients. We assessed the causal connection between them by graph theory and GCA. Compared to HCs, anti-NMDAR encephalitis patients showed aberrant topological properties in the left insula and abnormal causal connection both from and to the left insula. In summary, patients with anti-NMDAR encephalitis exhibit both functional brain impairment and post-injury compensation. The detected aberrations of the functional brain network structure contribute to better understanding of the neuropathological and physiological mechanisms of patients with anti-NMDAR encephalitis.

## Data Availability Statement

The raw data supporting the conclusions of this article will be made available by the authors, without undue reservation.

## Ethics Statement

The studies involving human participants were reviewed and approved by the First Affiliated Hospital of Guangxi Medical University Ethics Committee. The patients/participants provided their written informed consent to participate in this study.

## Author Contributions

CL: writing–original draft and experimental design. JL and QL: data curation. KS: investigation. XP: methodology. JZ: funding acquisition. All authors finally agreed to publish this manuscript.

## Conflict of Interest

The authors declare that the research was conducted in the absence of any commercial or financial relationships that could be construed as a potential conflict of interest.

## Footnotes

1 http://www.nitrc.org/projects/gretna/

2 http://www.fil.ion.ucl.ac.uk/spm/software/spm12/

3 http://www.restplusfmri.net
